# A Deep Learning Approach with Data Augmentation to Predict Novel Spider Neurotoxic Peptides

**DOI:** 10.3390/ijms222212291

**Published:** 2021-11-13

**Authors:** Byungjo Lee, Min Kyoung Shin, In-Wook Hwang, Junghyun Jung, Yu Jeong Shim, Go Woon Kim, Seung Tae Kim, Wonhee Jang, Jung-Suk Sung

**Affiliations:** 1 Department of Life Science, Biomedi Campus, Donnguk University-Seoul, 32, Dongguk-ro, Ilsandong-gu, Goyang-si 10326, Korea; blee.inf@gmail.com (B.L.); samantha1994@naver.com (M.K.S.); hiw9100@gmail.com (I.-W.H.); junghyunjj219@gmail.com (J.J.); shimyj12@gmail.com (Y.J.S.); gowoone@gmail.com (G.W.K.); 2 Life and Environment Research Institute, Konkuk University, 120, Neungdong-ro, Gwangjin-gu, Seoul 05029, Korea; stkim2000@hanmail.net

**Keywords:** deep learning, data augmentation, convolutional neural network, neurotoxic peptide prediction, spider transcriptome

## Abstract

As major components of spider venoms, neurotoxic peptides exhibit structural diversity, target specificity, and have great pharmaceutical potential. Deep learning may be an alternative to the laborious and time-consuming methods for identifying these peptides. However, the major hurdle in developing a deep learning model is the limited data on neurotoxic peptides. Here, we present a peptide data augmentation method that improves the recognition of neurotoxic peptides via a convolutional neural network model. The neurotoxic peptides were augmented with the known neurotoxic peptides from UniProt database, and the models were trained using a training set with or without the generated sequences to verify the augmented data. The model trained with the augmented dataset outperformed the one with the unaugmented dataset, achieving accuracy of 0.9953, precision of 0.9922, recall of 0.9984, and *F*1 score of 0.9953 in simulation dataset. From the set of all RNA transcripts of *Callobius koreanus* spider, we discovered neurotoxic peptides via the model, resulting in 275 putative peptides of which 252 novel sequences and only 23 sequences showing homology with the known peptides by Basic Local Alignment Search Tool. Among these 275 peptides, four were selected and shown to have neuromodulatory effects on the human neuroblastoma cell line SH-SY5Y. The augmentation method presented here may be applied to the identification of other functional peptides from biological resources with insufficient data.

## 1. Introduction

Spiders constitute the most diverse terrestrial invertebrate taxonomic group, which has evolved for >300 million years and adapted to various environmental conditions [1]. Such thriving was possible due to the venom production from venom glands that can be used both to defend against predators and hunt prey [2]. Cysteine-rich neurotoxic peptides in spider venom are known to affect the nervous system by targeting specific receptors, ion channels, and synaptic vesicle exocytosis [3,4,5,6]. The specific targeting property gives rise to many beneficial properties, such as insecticidal, pain control, and other pharmaceutical potentials [7,8,9,10,11]. For example, π-TRTX-Pc1a peptide from tarantula *Psalmopoeus cambridgei* has shown analgesic effects in vivo by inhibiting ASIC1a channel, and M-TRTX-Gr1a from the tarantula *Grammostola rosea* venom suppressed atrial fibrillation by inhibiting mechanosensitive channel. As the next-generation sequencing (NGS) technique became more easily accessible, there has been great effort to screen neurotoxic peptides from transcriptome data of spider venoms by identifying cysteine patterns and disulfide bond formation [12,13,14,15,16].

Although the biological data is accumulating, low accessibility due to the technical limitations for screening restricts the identification of neurotoxic peptides. The NGS technique generates genomic data implicating the complex interconnection of biological information. Since machine-learning algorithms are a powerful method when analyzing large and complex datasets, they may be suitable for utilizing the NGS data [17,18]. Conventional machine learning technique are also applied to predict protein–ligand binding affinity and epitope region with the physicochemical properties of peptides data [19,20]. Distinctively, deep learning enables the extraction of latent feature information from complex data that contributes to improved accuracy of the model prediction, such as annotating single-cell RNA-seq data, predicting the phosphorylation site of protein, identifying cancer subtypes, and functional prediction of peptides [21,22,23]. For example, research has been conducted to identify antimicrobial activity from peptide sequence by a convolutional neural network (CNN)-based model using multiple encoding methods [24]. However, the application of machine learning to the discovery of neurotoxic peptides is currently problematic because of insufficient data of neurotoxic peptides that draw unintentionally biased results. 

Here, a peptide data augmentation method was developed that enhances the performance of a deep learning model for neurotoxicity prediction. The augmentation was conducted by generating peptide sequences based on neurotoxic peptides and selecting the sequences homologous to the known neurotoxic peptides. When the models were trained with either augmented (AUG) or unaugmented (unAUG) datasets, the model trained with the AUG data outperformed those trained with the unAUG data, demonstrating that the augmentation method fully mimicked the features of neurotoxicity. Finally, novel potential neurotoxic peptides were discovered from the best performed model in the simulation dataset among the transcriptome of an endemic spider of South Korea, *Callobius koreanus* (*C. koreanus*). Our results suggest that the augmentation strategy improving identification of novel neurotoxic peptides can broaden the opportunity to investigate functional bioactive compounds.

## 2. Results

### 2.1. Application of an Augmentation Method to the Preparation of the Dataset for Model Training

**Figure 1:**
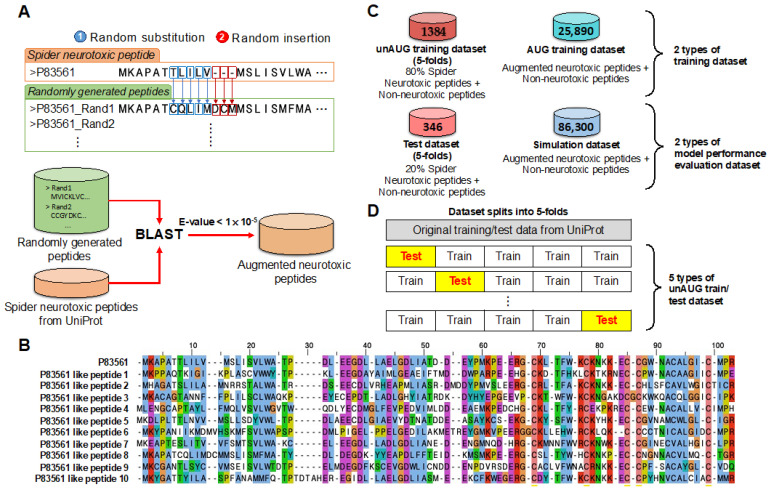
Data preparation overview. (**A**) Neurotoxic peptide data were augmented by using known peptides. The sequences were generated by random substitution and insertion of amino acids, and peptides under the *E*-value of 1 × 10^−5^ were selected by BLAST. (**B**) An example of a known neurotoxic peptide (P83561) and the derived AUG peptides. (**C**) Four types of datasets, two for training, and two for performance testing were prepared to evaluate the validity of the AUG neurotoxic peptides. (**D**) unAUG training and test data included neurotoxic and non-neurotoxic peptides from the UniProt. The data were split into 5-fold, of which one fold was selected for the test, and the others for model training.

For a deep learning model to avoid overfitting and class imbalance problems, a large amount of data and uniform class distribution is indispensable. Peptide sequences for training and validation were collected from the UniProt database (Figure S1A), yet the number of neurotoxic peptides was insufficient compared to the non-neurotoxic peptides. Accordingly, we augmented the data by randomly substituting or inserting arbitrary amino acids based on known neurotoxic peptides (Figure 1A). The generated sequences were selected for augmentation only when the sequence showed homology with known neurotoxic peptides from the UniProt by Basic Local Alignment Search Tool (BLAST) (*E*-value < 1 × 10^−5^) [25]. The example of the sequence alignment between neurotoxic and augmented neurotoxic peptides was shown (Figure 1B).

To verify the augmented peptides, we organized four types of datasets (Figure 1C). The unAUG training and AUG training datasets, individually containing only known or augmented neurotoxic peptides, were created to confirm whether the augmented data sufficiently expresses the characteristics of neurotoxic peptides by the differences in data organization. The test and simulation datasets were organized to assess model prediction performance. The known peptides were distributed into unAUG training and test datasets for 5-fold cross-validation to avoid data selection bias when evaluating model performance (Figure 1D). Considering that the number of neurotoxic peptides in test data was 173, which was insufficient to measure model performance, the simulation data comprised adequate AUG data that were not included in AUG training datasets (Figure 1D). Finally, we prepared two types of the training datasets—unAUG and AUG—to train the model, and two types of datasets—test and simulation datasets—to evaluate the model performance (Table S1).

### 2.2. Selection of an Optimized Deep Learning Model for the Identification of Neurotoxic Peptides 

**Figure 2:**
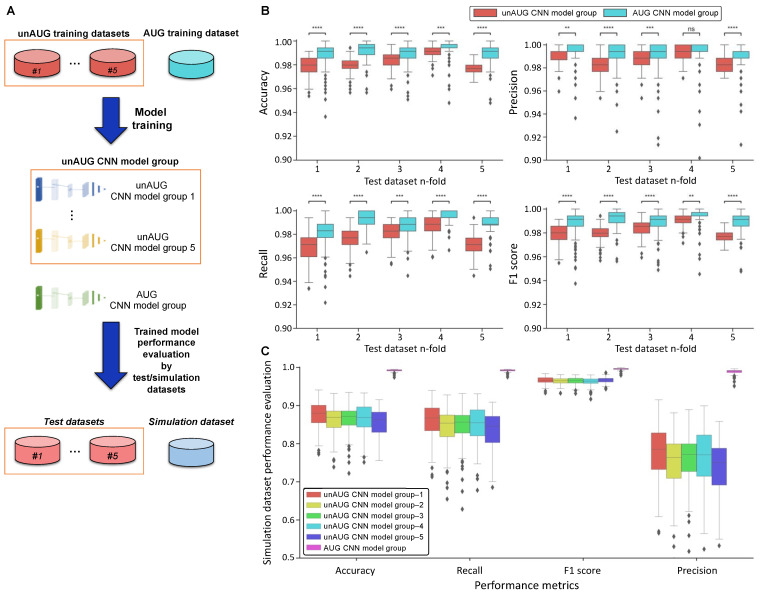
Prediction results of the CNN models using test and simulation datasets. (**A**) CNN models were trained by AUG and unAUG training datasets. Trained model performances were evaluated based on 5-fold of test datasets and a simulation dataset. (**B**) The performance results of test dataset prediction are represented in boxplots. The prediction performances of unAUG and AUG CNN models were compared by four performance metrics of accuracy, precision, recall, and *F*1 scores (** *p <* 0.01, *** *p <* 0.001, **** *p* ≤ 0.0001). (**C**) Boxplots showing simulation dataset prediction performance results. The prediction performance of the models was compared with the above four performance metrics.

To assess whether the AUG dataset is more effective than the unAUG dataset, a comparative analysis of deep learning models was conducted along with the verification of the augmentation method. Among the various deep learning architectures, we selected the convolutional neural network (CNN). For the comparison between the datasets, various model hyperparameters were applied to each training dataset. A total of 864 CNN models were obtained by 6 training data types of 5-fold unAUG datasets and an AUG dataset, together with 2 learning rates, 24 model structures, and 3 iterative learning (Table S2). Two trained model groups—unAUG and AUG—were generated, where the unAUG CNN model group was divided into groups 1 to 5 according to dataset folds for 5-fold cross-validation (Figure 1D and Figure 2A). 

To compare the prediction performance by the training dataset, model prediction performances were measured by four performance metrics—accuracy, precision, recall, and *F*1 score—and the statistical analyses between the unAUG and AUG CNN model groups were performed. In the test dataset, the AUG CNN model group excelled in every metric, which showed statistical significance with *p*-value under at least 0.05 except only precision with the 4-fold test dataset (Figure 2B). The unAUG CNN model group showed poor performance on recall, misclassifying true neurotoxic peptides as non-neurotoxic peptides more abundantly. Furthermore, the AUG CNN model group achieved better performance on neurotoxic peptide classification in the simulation dataset (Figure 2C).

Further analysis was conducted based on the difference in model performance by hyperparameters. The model architectures in the unAUG CNN model group did not significantly affect the prediction performance of test and simulation datasets (Figures S2 and S3). In the case of the AUG CNN model group, the precision parameter of simulation prediction was increased with the number of conv-pool layers. The architecture with four conv-conv-pool layers showed improved performance over the other structures (Figure S5A), whereas the other structural differences did not have any significant effect on the performance (Figures S4 and S5B–D). 

Altogether, the CNN models trained by the AUG dataset showed better performance compared with the other models, strongly suggesting that the AUG dataset sufficiently represented characteristics of neurotoxic peptides. The optimized models from each model group were selected based on the *F*1 score of the simulation dataset. The cases that showed the best performance were the hyperparameters of no. 16 and no. 2 from the AUG and unAUG CNN model groups (Table 1), respectively, which were chosen for screening neurotoxic peptides.

### 2.3. Screening C. koreanus Transcriptome for Neurotoxic Peptides

**Figure 3:**
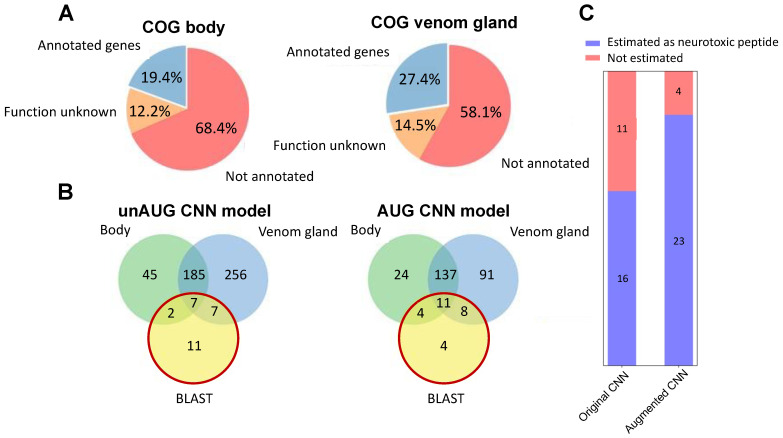
Transcriptome analysis of *C. koreanus* and comparison of estimation results from the CNN models and the BLAST. (**A**) The annotation results of COG from the body (**left**) and the venom gland (**right**) were shown in pie charts. (**B**) Estimated neurotoxic peptides by the unAUG model (**left**) and the AUG model (**right**) were presented along with the BLAST results. (**C**) The number of the putative neurotoxic peptides predicted from the BLAST search was larger in the AUG model than in the unAUG model.

In order to identify neurotoxic peptides via the two obtained CNN models, *C. koreanus*, an endemic spider in South Korea, was selected for the transcriptome data generation and analysis. The venom gland was separated from the body, and each venom gland and the rest of the body was subjected to de novo assembly (simply stated as “body” hereupon). A total of 151,080 transcripts and 21,214 protein-coding genes were identified (Table S3); 15,411 and 10,116 genes were found to be expressed in the body and venom gland, respectively. The number of differentially expressed genes (DEGs) between *C. koreanus* body and venom gland was individually found to be 4962 and 5275 genes (Figure S8). By using the egg-NOG mapper, the protein-coding genes among *C. koreanus* transcripts were sought for gene ontology (GO) and clusters of orthologous groups (COG) analyses. The results showed that more than half of the genes were not annotated or assigned to unknown function in the COG (Figure 3A and Figure S9) as well as in the GO terms (Figures S10 and S11). Additionally, the body showed less annotation, as the overall functionality of the transcript data was rarely known. 

We searched for the putative neurotoxic peptides among the *C. koreanus* transcripts via the two selected CNN models and BLAST search after translating the predicted coding regions. BLAST is a conventional and popular way to predict function based on protein sequence, which stems from homology analysis [26]. Thus, the BLAST search was conducted for comparison with the predicted results of the CNN models to confirm whether the selected models can discover the novel neurotoxic peptides not identified in a conventional way. A total of 27 peptides from *C. koreanus* showed homology with the known spider neurotoxic peptides by BLAST (Table 2). When we screened *C. koreanus* transcriptome data by two CNN models, each AUG and unAUG CNN model estimated 275 and 628 putative neurotoxic peptides (Figure 3B). The overlapping peptides with the BLAST result were 23 peptides and 16 peptides from the AUG and the unAUG CNN models (Figure 3C and Table 2). Although the number of estimated peptides was larger in the unAUG model, the AUG model showed a higher overlapping ratio with the BLAST results. The AUG CNN model captured every peptide that showed homology with *E*-value under 1 × 10^−8^ from the BLAST result. In conclusion, it was suggested that the AUG CNN model outperformed the unAUG model in identifying putative neurotoxic peptides. Regarding the results from the AUG model, a total of 252 sequences lacking homology with the known-neurotoxic peptides were identified, of which 32 and 46 were significantly differentially expressed in the body and venom gland, respectively (Figure S12).

### 2.4. Experimental Investigation of Selected Neurotoxic Peptides

To further validate the capability of the AUG CNN model in predicting neurotoxicity, we selected four putative neurotoxic peptides for further analysis and experimentation (Table 3). Among the selected peptides, c136163, c43972, and c68875 were novel, and c62771 was the only peptide that showed homology with known neurotoxic peptides. Notably, c136163 was predicted to be expressed only in the venom gland. Before the experimental assessment, the functional regions of the peptides were determined and synthesized because the full sequences were lengthy for synthesis. First, four putative peptides were first predicted of mature peptide region by SignalP and SpiderP server, without the signal and propeptide region (Table S4) [27,28]. As the cysteine-rich neurotoxic peptides possess characteristic structural motifs or features, the secondary structure and disulfide bond were analyzed by XtalPred Server and Disulfind tool [29,30]. The regions predicted to have disulfide bonds that match the pattern of the known neurotoxins and/or secondary structure were selected to be functional from the putative neurotoxic peptides and were synthesized for the experimental evaluation of neurotoxic potential (Table 4) [31,32,33]. 

**Figure 4:**
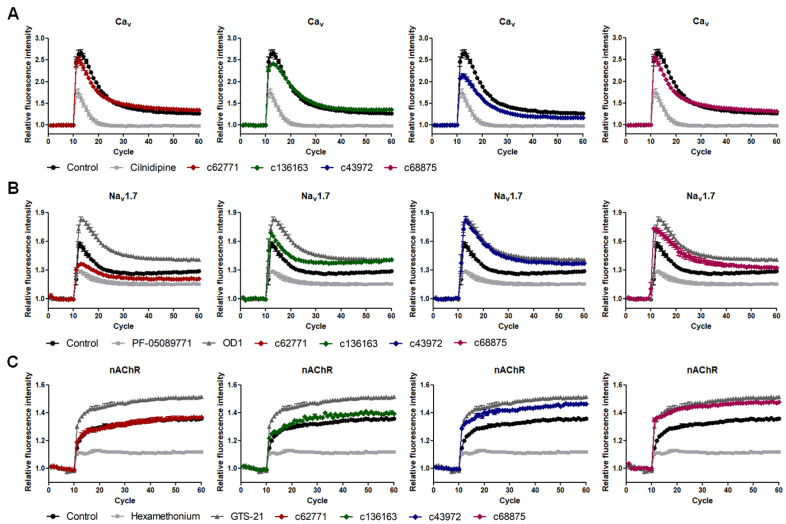
Modulatory effects of predicted peptides on the ion channel activity. Each of the four peptides from the AUG model prediction showed either activation or inhibition on specific ion channel subtype. Peptides were treated with the final concentration of 10 μM. (**A**) Peptide c43972 had an inhibitory effect on Ca_v_ when compared with the L-/N-type calcium channel inhibitor cilnidipine. (**B**) Peptide c62771 reduced the activity of Na_v_1.7 channels, whereas c136163, c43972, and c68875 activated the channel. (**C**) The nAchR were activated when treated with c43972 and c68875.

Human neuroblastoma cell line SH-SY5Y was used as the cell line maintains various properties of neurons in culture. As the cell line stably expresses functional L-/N-type Ca_v_ channels, Na_v_1.7 channels, and nicotinic acetylcholine receptors (nAChRs), we targeted these channels for investigating the modulatory activity of the peptides [34,35,36,37]. Specific activators and inhibitors were selected to be used as positive and negative controls for the comparison. The intracellular calcium ion influx was measured via fluorescent dye fluo-4 that binds with calcium ions in a live cell. The relative fluorescence was compared among that of the inducer treatment and that of the inhibitor, activator, or 10-μM peptide treatment (Materials and Methods Section 4.6). The maximum increase in the signal was evaluated as to whether the peptide significantly modulated the ion channel activity. It was suggested that all of the four synthesized peptides have neuromodulatory effects on the targeted ion channel or receptor (Figure 4). c62771 exhibited an inhibitory effect on the Na_v_1.7 channel, while c136163 showed a mild increase on the same ion channel. In the case of c43972 and c68875, the peptides activated the activity of the Na_v_1.7 channel and nAChR, comparable to the activators OD1 and GTS-21. The results showed that the putative neurotoxic peptides predicted by the AUG CNN model are potentially neurotoxic. 

## 3. Discussion

The advancement of deep learning and data production technologies has led to the grafting of deep learning technologies onto various fields of biology [38]. However, there are specific fields that face difficulty in data accumulation, which are often confronted with “the curse of dimensionality” due to small amounts of data [39]. Neurotoxic peptides from spiders are one such case where the dataset is insufficient, making it unfeasible to train machine learning algorithms for classifying neurotoxic peptides. In this study, we developed a new data augmentation method that successfully generates and selects biologically significant data for neurotoxic peptides. 

Data augmentation is a method that enables the enlargement of data diversity without collecting new data. Recent studies showed an automated search for best augmentation policy in image classification models, where it confirmed the model performance increase in deep learning algorithms [40,41]. These cases show that using the appropriate augmentation methods can bring out significant generalization improvements, leading to a better application of the trained model onto unseen data drawn from the same distribution. The application of the general methodology of natural language processing is difficult since the peptide sequences are string data represented with amino acid residues. Generative models, including generative adversarial network (GAN) and variational autoencoder (VAE), are often used for peptide sequence augmentation that lacks data; however, such technique has a limitation in that the model must be trained for each application [42,43].

To develop a data augmentation technique for general peptides without additional training steps, a BLAST-based augmentation method was implemented. BLAST is a major tool that is actively used in biological fields that provides information on similarity and homology among known sequences. The Generalized functional prediction was possible since BLAST predicts functionality-based sequence similarity. The augmented sequences were generated while retaining the distribution of amino acid residues, and then the generated sequences were screened and selected via BLAST. Thus, we applied this method to identify neurotoxic peptides derived from spiders.

In order to confirm the validity of the AUG dataset, we trained the CNN models with each of the AUG and unAUG training datasets and then evaluated the models by using the test and simulation datasets. We organized a limited environment with a small amount of data by using spider-specific 865 peptides. To focus on the productivity of the dataset, model hyperparameters were identically applied to both training datasets. The prediction results of the test and the simulation dataset indicated the classification performance of the known peptide dataset and the degree of generalization. In this regard, the AUG CNN model group outperformed in known and simulation datasets, suggesting that our augmentation method has extracted biological features from the known neurotoxic peptides successfully. The optimized model from each model group was selected for further analysis using the actual example of the spider transcriptome.

The transcriptomic data from *C. koreanus* were screened for the predicted neurotoxic peptides by using each optimized model. The BLAST resulted in 27 peptides that showed significant similarities with spider neurotoxic peptides. The prediction results by the AUG CNN model contained more overlapping sequences from BLAST results than those of the unAUG model, and the comparison confirmed the effectiveness of the AUG dataset. Four peptides were selected to evaluate their neurotoxic potentials. Among these peptides, 1 was predicted via both models, BLAST search and DEG results (c62771); 1 peptide was predicted only from both models (c68875); and 2 peptides were predicted only via the AUG model (c136163 and c43972). The experiments were conducted to determine the modulatory effects of the peptides on ion channels, L- or N-type Cav, Nav 1.7, and nAchR by measuring the calcium ion influx. Four peptides were shown to possess modulatory activity on specific subtypes of ion channels, concurring the results of the AUG model prediction. As the AUG CNN model successfully discovered potential neurotoxic peptides, it is suggested that the augmented neurotoxic peptide data contributed to finding additional two novel neurotoxic peptides that were not identified by the unAUG model or BLAST search.

Various research is being conducted to utilize the neurotoxic peptides advantageously, and spider venom is a major target as it possesses them in abundance. Up to this point, the number of known neurotoxic peptides in the UniProt database may be insufficient for deep learning training. Thus, we developed a peptide data augmentation method containing latent representation of the biological information. We successfully demonstrated that AUG data mimicked the known neurotoxic peptides, suggested by the actual performance of the AUG CNN model. It is expected to be more effective for model training when the known and augmented peptides are simultaneously applied in other types of peptide data. Further, as the model performance improved using the traditional CNN model; we believe that incorporating the peptide data augmentation method into the state-of-the-art models may even boost the prediction performance. The result may provide a useful method for peptide data augmentation and alleviate the limitations of data deficiency, aiding the research on deep learning applications in biology.

## 4. Materials and Methods

### 4.1. Data Preparation

The peptide data for the training of CNN models were obtained from the UniProt database [44] (Figure S1A). The sequences of the neurotoxic and non-neurotoxic peptides were obtained by using the keywords “spider AND neurotoxin” and “NOT neurotoxin” from the database, respectively, and the peptides of 50–300 amino acids were selected. The augmented neurotoxic peptides were generated to increase the size of the model training dataset (Figure S1B). The sequences were created by random substitution and insertion of amino acids of the known neurotoxic peptides. For each amino acid in the existing neurotoxic peptides, random substitution was performed with a 50% chance. When the random substitution was performed, the amino acid was replaced with an amino acid with a side chain of similar physiochemical properties by a 60% chance. Random insertion was performed with a 10% chance by selecting an arbitrary amino acid. These sequences were selected by BLAST v2.9.0 with a cutoff *E*-value of 1 × 10^−5^. 

Four types of datasets were prepared (Supplementary Material; Figure S1C); the known neurotoxic peptides comprised the unAUG training and test datasets, whereas the generated sequences comprised the AUG training and simulation datasets (Figure 1C). The known sequences were randomly partitioned into unAUG training and test datasets for 5-fold cross-validation (Figure 1D). In each dataset, the known non-neurotoxic peptides were randomly selected and included in equal amounts as neurotoxic sequences. The known non-neurotoxic peptides were included as the equal amount of each dataset’s neurotoxic sequences. The data were transformed using a one-hot-encoding method by converting peptide sequences into a two-dimensional array. 

### 4.2. Selection of the CNN Model

The training data were separated into training and validation data to obtain an optimized CNN model. Hyperparameters of different model architectures, learning rates, and training data were used to evaluate CNN models. Twenty-three architectures of the CNN model (Table S1) were trained with two learning rates (0.005 and 0.0001), Adam optimizer and training data (5-fold training and AUG training datasets), which were repeated three times each. Softmax function was used at the final node to identify the neurotoxic peptides as follows:

$$softmax\left(x_{i}\right)=\frac{e^{x_{i}}}{\sum_{j=1}^{2}e^{x_{j}}}$$

for i=1,2—where 1 is for non-neurotoxicity class and 2 for neurotoxicity class. 

The performances of the trained models were measured by accuracy, precision, recall, and *F*1 score as follows:

(1) $$Accuracy=\frac{TP+FP}{TP+FP+TN+FN}$$

$$Precision=\frac{TP}{TP+FP}$$

$$Recall=\frac{TP}{TP+FN}$$

$$F1=2\frac{Precision\times Recall}{Precision+Recall}$$

where *TP* stands for a true positive number, *TN* for a true negative number, *FP* for a false negative number, and *FN* for a false positive number. The best-performing models from each hyperparameter were acquired by the lowest *F*1 score of the validation dataset.

The models trained with the unAUG training dataset and AUG training dataset were grouped as the unAUG CNN model group and AUG CNN model group, respectively. The significance of the difference between the two groups was determined using the paired *t*-test. Finally, we selected the best-performing model from each of the unAUG CNN and AUG CNN models according to the lowest *F*1 score from the simulation data.

### 4.3. Preparation of C. koreaus Samples

The spider *C. koreanus* was collected from Chungbuk, Korea. The venom glands of the spider were separated from the chelicerae and stored at −80 °C after washing with phosphate-buffered saline. TRIzol Reagent (Life Technologies, Grand Island, NY, USA) was used for extracting total RNA, which was subsequently used for NGS (Theragen Etex Bio Institute, Suwon, Korea). The sequencing was performed in triplicate for both venom gland and body, producing 6 data pools. 

### 4.4. De Novo Assembly and Functional Annotation of the Transcriptome

Paired-end sequencing reads of cDNA libraries (101bp) were generated using a NovaSeq6000 instrument (Illumina, San Diego, CA, USA), and then verified for their sequence quality by using FastQC v 0.10.0. For data preprocessing, the low-quality bases and adapter sequences among the reads were trimmed using Trimmomatic v0.3225. The trimmed reads were assembled using Trinity (strand-specific option: --SS_lib_type RF) [45]. The Trinity program was utilized for de novo transcriptome assembly to generate unigenes. The unigenes were further processed for read alignment and abundance estimation by using Bowtie and RSEM [46,47]. The expression level of each unigene was calculated using the Fragments Per kilobase of exon per Million mapped fragments (FPKM) method. We filtered contigs out when at least one sample read count was zero in one group (body or venom gland) or lowly expressed (average FPKM ≤ 1) in both groups. The transcripts detected in the body or venom gland were classified into GO and COG by using eggnog-mapper with the *E*-value cutoff of 1 × 10^−3^ [48] (Figure S1F). The read count data of filtered genes were normalized by Relative Log Expression normalization with DESeq2, which includes the nbinomTest function tests for differential expression [49]. The output-printed fold change and *p*-value, and FDR values were corrected by the Benjamini–Hochberg procedure. DEGs were determined by |fold change| ≥ 2 and *p*-value < 0.05. For DEGs, hierarchical clustering analysis was performed with a complete linkage method and Euclidean distance as a measure of similarity. 

### 4.5. Identification of the Neurotoxic Peptides in C. koreanus 

TransDecoder v5.3.0 was used to extract the peptide-coding regions from the reference assembly [50]. Putative peptide data were obtained from both groups, body and venom gland, separately. Neurotoxic peptides from *C. koreanus* were predicted by the unAUG and AUG CNN models and BLAST (Figure S1G). The BLAST result was used as a baseline to compare the performances of the CNN models. We searched the UniProt spider neurotoxic peptide data for peptides homologous to *C. koreanus* peptides by using BLAST with a cutoff *E*-value of 1 × 10^−5^. The overlapping sequences between our CNN models and BLAST results were identified.

### 4.6. Reagents

Dulbecco’s modified eagle medium fetal bovine serum (FBS), penicillin, and streptomycin (PS) were purchased from Gibco (Grand Island, NE, USA). Quanti-Max WST-8 Cell Viability assay kit (Biomax, Seoul, Korea) and Fluo-4 NW Calcium Assay Kit (Invitrogen, Carlsbad, CA, USA) was obtained for in vitro assays. Selective inducers, agonists, and antagonists against Ca_v_, Na_v_1.7, and nAChR were prepared for the intracellular calcium ion measurement. Hank’s balanced salt solution (Gibco) with 20 mM N-2-hydroxyethylpiperazine-N-2-ethane sulfonic acid was used as an assay buffer and every reagent was diluted in the buffer according to their final concentration. For inducers, 5-mM calcium chloride (Sigma-Aldrich, St. Louis, MO, USA), 90-mM potassium chloride (Sigma-Aldrich), 50-μM veratridine (Abcam, Cambridge, MA, USA), and 30-μM nicotine (Sigma-Aldrich) were prepared for Ca_v_, Na_v_1.7, and nAChR, individually. L-/N-type Ca_v_ blocker cilnidipine, Na_v_1.7 channel blocker PF-05089771, and nAChR antagonist hexamethonium bromide were purchased from Sigma-Aldrich, and the final concentration used for assays were 15 μM, 50 nM, and 100 μM, respectively. Na_v_1.7 activator OD1 (R&D Systems, Minneapolis, MN, USA) and α7 nAChR agonist GTS-21 dihydrochloride (Abcam) were each treated with the final concentration of 20 nM and 10 μM. The peptides used in assays were synthesized by BioStem (Ansan, Gyeonggi, Korea), with purity >95% and verified by mass spectroscopy and high-performance liquid chromatography.

### 4.7. Cell Culture and Cell Viability Assay 

Human neuroblastoma SH-SY5Y cells were purchased from the American Type Culture Collection (Manassas, VA, USA) and maintained in DMEM supplemented with 10% FBS and 1% PS. The cells were cultured under a humidified atmosphere at 37 °C with 5% CO_2_. Before further investigation, cell viability assay was conducted to test the cytotoxicity of the peptides against SH-SY5Y. The cells were seeded on a 96-well plate and cultured for 24 h. After the cells were treated with the peptides (1, 5, or 10 μM) for 24 h, WST-8 solution was added to each well and then incubated for 1 h. The absorbance was measured at 450 nm using a microplate reader (Molecular Devices, Sunnyvale, CA, USA). All the experiments were conducted in triplicate, and the results were expressed as mean ± SEM. The statistical significance of the data was evaluated by one-way ANOVA followed by Tukey’s post-test. The results are shown in Figure S13.

### 4.8. Intracellular Calcium Ion Measurement

Fluo-4 AM is a cell-permeable Ca^2^+ indicator that its fluorescence enhances upon intracellular calcium ion binding. To investigate the modulatory effect of the peptides on ion channels, the Fluo-4 NW kit was used according to the manufacturer’s protocol. In brief, SH-SY5Y cells were seeded on 96-well black plates and cultured for 48 h. The background fluorescence was measured after the dye was incubated at 37 °C for 30 min followed by an additional 30 min at room temperature. The cells were then treated with an inhibitor or activator of each ion channel or 10-μM peptide for 10 min to evaluate ion channel activity. Fluorescent responses were measured at the excitation and emission wavelengths of 470–495 nm and 515–575 nm, respectively, by using the Infinite F200 Pro multimode microplate reader (Tecan, Männedorf, Switzerland). The inducer was injected after measurement of 10 cycles, and the following 60 cycles were continued. The changes in fluorescence intensity were normalized to the baseline and plotted for each ion channel using GraphPad Prism 5.03 (GraphPad Software, La Jolla, CA, USA). All the experiments were conducted in triplicate, and the results were expressed as mean ± SEM.

## 5. Conclusions

The augmentation method in this study successfully mimicked the features of neurotoxic peptides. The augmented data improved the prediction performance of the deep learning model, leading to the discovery of novel peptides. The AUG CNN model predicted the putative neurotoxic peptides in *C. koreanus* transcriptome, and four selected sequences showed neuromodulatory potency. Since the augmentation method was based only on the peptide sequences, it may be applied to the development of other prediction models using peptide data without limitations.

## Figures and Tables

**Table 1:** The prediction performances of the optimized models on simulation data.

	Accuracy	Precision	Recall	*F*1
unAUG CNN model	0.9410	0.9149	0.9653	0.9395
AUG CNN model	0.9953	0.9922	0.9984	0.9953

**Table 2:** The list of peptides estimated from CNN models overlapping with the BLAST result.

	*C. koreanus* ID	Uniprot Accession ID	*E*-Value	Bitscore	CNN Prediction Results	
					unAUG CNN Model	AUG CNN Model
1	c142900	P15969	3.74 × 10^−43^	134.42	Neurotoxin	Neurotoxin
2	c66652	P15969	1.99 × 10^−40^	127.487	Neurotoxin	Neurotoxin
3	c47691	B3EWT5	7.55 × 10^−37^	118.242	-	Neurotoxin
4	c7268	P15969	1.51 × 10^−34^	112.079	Neurotoxin	Neurotoxin
5	c63588	Q5Y4U3	7.15 × 10^−32^	105.916	-	Neurotoxin
6	c64685	B6DCU0	3.00 × 10^−27^	93.9745	Neurotoxin	Neurotoxin
7	c14525	P15969	5.44 × 10^−26^	90.1225	Neurotoxin	Neurotoxin
8	c103362	B6DD31	5.99 × 10^−19^	71.633	Neurotoxin	Neurotoxin
9	c68025	P15969	1.49 × 10^−17^	67.3958	Neurotoxin	Neurotoxin
10	c70375	Q5Y4U2	4.29 × 10^−15^	62.003	-	Neurotoxin
11	c48731	Q5Y4U3	8.59 × 10^−13^	54.6842	Neurotoxin	Neurotoxin
12	c62771	Q5Y4U4	5.57 × 10^−12^	51.9878	Neurotoxin	Neurotoxin
13	c68135	B3EWT5	4.28 × 10^−10^	46.595	Neurotoxin	Neurotoxin
14	c12324	B3EWT5	7.29 × 10^−10^	46.595	-	Neurotoxin
15	c63710	P83303	1.98 × 10^−8^	41.5874	Neurotoxin	Neurotoxin
16	c68692	B3EWT5	6.73 × 10^−8^	40.817	-	-
17	c31828	B3EWT5	1.94 × 10^-7^	38.891	Neurotoxin	Neurotoxin
18	c67995	P0C2S9	2.07 × 10^−7^	38.891	Neurotoxin	-
19	c61830	B3EWT5	2.46 × 10^−7^	38.5058	-	Neurotoxin
20	c72098	Q8MTX1	2.89 × 10^−7^	38.891	Neurotoxin	Neurotoxin
21	c62649	Q8MTX1	5.47 × 10^−7^	38.5058	-	Neurotoxin
22	c50230	Q8MTX1	6.96 × 10^−7^	38.5058	-	-
23	c68303	B3EWT5	1.14 × 10^−6^	37.7354	-	Neurotoxin
24	c65952	P59367	2.25 × 10^−6^	36.1946	Neurotoxin	Neurotoxin
25	c33223	Q5Y4U4	6.97 × 10^−6^	35.039	Neurotoxin	Neurotoxin
26	c31543	B3EWT5	9.04 × 10^−6^	35.4242	-	Neurotoxin
27	c51710	B3EWT5	9.08 × 10^−6^	34.6538	-	-

**Table 3:** Selected neurotoxic peptides predicted with neuromodulatory effects.

Name	BLAST Search		Body	Venom Gland	DEG Result		CNN Prediction Results	
	UniProt Accession ID	*E*-Value			Fold Change	*p*-Value	unAUG CNN Model	AUG CNN Model
c62771	Q5Y4U4	5.57 × 10^−12^	Expressed	Expressed	1.52	4.00 × 10^−1^	Neurotoxin	Neurotoxin
c136163	-	-	Expressed	-	-	-	-	Neurotoxin
c43972	-	-	Expressed	Expressed	−3.09	3.00 × 10^−4^	-	Neurotoxin
c68875	-	-	Expressed	Expressed	−72.3	2.00 × 10^−7^	Neurotoxin	Neurotoxin

**Table 4:** Target peptide region expected of neuromodulatory function.

Name	Secondary Structure	Cysteine Distribution/Disulfide Bond Prediction	Length
c62771	β-sheet	SCIRRSASCDHRPSDCCFNSSCRCNLWGTNCRCQRAGLFQKWGK [C1–C5, C2–C6, C3–C7, C4–C8]	44
c136163	β-sheet	KCRLEGCKSRTRVCVKCQMYLCIMKNNCF [C1–C4, C2–C5, C3–C6]	29
c43972	β-sheet	WCSCGLSKKQPFCDGSHINHPKKLQPVRFNPPKDGRFLLCRCKQTNNRPYCD [C1–C4, C2–C5, C3–C6]	52
c68875	α-helix	GRRGRRQRCSSLLRNWERCDRRNQCPCGAGL -	31

## Data Availability

The neurotoxic peptide screening model and peptide data augmentation code developed in this study are freely available at GitHub (https://github.com/bzlee-bio/NT_estimation (accessed on 11 November 2021)). under a GPL-v3 license. The processed *C. koreanus* transcriptome data can be downloaded from NCBI (GSE158565).

## Supplementary Materials

The following are available online at https://www.mdpi.com/article/10.3390/ijms222212291/s1. Figure S1: Research workflow; Figure S2: Prediction results from the unAUG CNN model with the test data; Figure S3: Prediction results from the unAUG CNN model with the simulation data; Figure S4: Prediction results from the AUG CNN model group with the test data; Figure S5: Prediction results from the AUG CNN model with the simulation data; Figure S6: Preprocessing of *C. koreanus* transcriptomic data; Figure S7: Reproducibility among *C. koreanus* samples; Figure S8: DEG analysis of *C. koreanus* transcriptomic data; Figure S9: Functional annotation of the clustal of orthologous groups (COGs) from *C. koreanus* peptide-coding genes; Figure S10: Functional annotation of gene ontology (GO) from *C. koreanus* peptide-coding genes; Figure S11: Distribution of the level-2 GO terms of *C. koreanus* peptide-coding genes; Figure S12: Comparison of *C. koreanus* transcriptome between the DEGs and prediction results from the AUG CNN model; Figure S13: Evaluation of cytotoxicity of the peptides against human neuroblastoma cell line; Table S1: Dataset information; Table S2: CNN model hyperparameters; Table S3: Genomic statistics of *C. koreanus*; Table S4. The predicted mature peptide region of the selected peptides.

## Abbreviations

The following abbreviations are used in this manuscript:

AUG	Augmented
BLAST	Basic Local Alignment Search Tool
CNN	Convolutional Neural Network
COG	Clusters of Orthologous Groups
*C. koreanus*	*Callobius koreanus*
DEGs	Differentially Expressed Genes
GAN	Generative Adversarial Network
nAChRs	Nicotinic Acetylcholine Receptors
NGS	Next-generation sequencing
unAUG	Unaugmented
VAE	Variational Autoencoder
