# Supplementary material for: A Deep Learning Approach with Data Augmentation to Predict Novel Spider Neurotoxic Peptides
Source: Int J Mol Sci. 2021 Nov 13;22(22):12291. doi: 10.3390/ijms222212291 (PMC8619404; doi:10.3390/ijms222212291)
Supplement: Supplementary file 1 [file ijms-22-12291-s001.zip › ijms-1449452-supplementary.pdf]

Table S1. Dataset information.

	unAUG training dataset (5-fold)	Test dataset (5-fold)	AUG training dataset	Simulation dataset
Neurotoxic peptide data from Uniprot	692	173	-	-
Augmented neurotoxic peptide data	-	-	12,945	43,150
Non-neurotoxic data from Uniprot	692	173	12,945	43,150
Total	1384	346	25,890	86,300

Table S2. CNN model hyperparameters.

No.	Model shape	Convolutional layer		Pooling layer		Fully connected layer
		Filter size	Kernel size	Pool size	Strides	Node shape
1	2 conv-pool 1 dense	8, 16	3,3	3,3	2	300
2	2 conv-conv-pool 1 dense	8, 16	3,3	3,3	2	300
3	2 conv-pool 1 dense	8, 16	5,5	5,5	2	300
4	2 conv-conv-pool 1 dense	8, 16	5,5	5,5	2	300
5	2 conv-pool 3 dense	8, 16	3,3	3,3	2	500-300-100
6	2 conv-conv-pool 3 dense	8, 16	3,3	3,3	2	500-300-100
7	2 conv-pool 3 dense	8, 16	5,5	5,5	2	500-300-100
8	2 conv-conv-pool 3 dense	8, 16	5,5	5,5	2	500-300-100
9	3 conv-pool 1 dense	8, 16, 32	3,3	3,3	2	300
10	3 conv-pool 1 dense	8, 16, 32	5,5	5,5	2	300
11	3 conv-conv-pool 1 dense	8, 16, 32	5,5	5,5	2	300
12	3 conv-pool 3 dense	8, 16, 32	3,3	3,3	2	500-300-100
13	3 conv-pool 3 dense	8, 16, 32	5,5	5,5	2	500-300-100
14	3 conv-conv-pool 3 dense	8, 16, 32	5,5	5,5	2	500-300-100
15	4 conv-pool 1 dense	8, 16, 32, 64	3,3	3,3	2	300
16	4 conv-conv-pool 1 dense	8, 16, 32, 64	3,3	3,3	2	300
17	4 conv-pool 1 dense	8, 16, 32, 64	5,5	5,5	2	300
18	4 conv-conv-pool 1 dense	8, 16, 32, 64	5,5	5,5	2	300
19	4 conv-pool 3 dense	8, 16, 32, 64	3,3	3,3	2	500-300-100
20	4 conv-conv-pool 3 dense	8, 16, 32, 64	3,3	3,3	2	500-300-100
21	4 conv-pool 3 dense	8, 16, 32, 64	5,5	5,5	2	500-300-100
22	4 conv-conv-pool 3 dense	8, 16, 32, 64	5,5	5,5	2	500-300-100
23	3 conv-conv-pool 1 dense	8, 8, 16, 16, 32, 32	3,3	3,3	2	300
24	3 conv-conv-pool 3 dense	8, 8, 16, 16, 32, 32	3,3	3,3	2	500-300-100

Table S3. Genome statistics of *C. koreanus*.

	Callobius koreanus
Transcripts	151,080
Number of candidate peptides	21,214
GC content	35.15%
N50	731
ORF predicted transcript	21,214
Number of ORF	22,258
Expressed gene in body	15,411
Expressed gene in venom gland	10,116

Table S4. The predicted mature peptide region of the selected peptides.

Name	Mature peptide sequence
c62771	SCIRRSASCDHRPSDCCFNSSCRCNLWGTNCRCQRAGLFQKWGK
c136163	SEEDEPPKKQRKVISQPVKEVRLDNVGHVPKFMDDKNASKCRLEGC KSRTRVCVKCQMYLCIMKNNCFLKYHTQ
c43972	IPIYITYAWCSCGLSKKQPFCDGSHINHPKKLQPVRFNPPKDGRFLLC RCKQTNNRPYCDLSHVKTFIPESLRKALKIKL
c68785	VEETTTDSTIEPSSSTTQPPPVSCGSQTCRANECCIQGRRGRRQRCSSLL RNWERCDRRNQCPCGAGLTCRAIRRRSYCRPQ

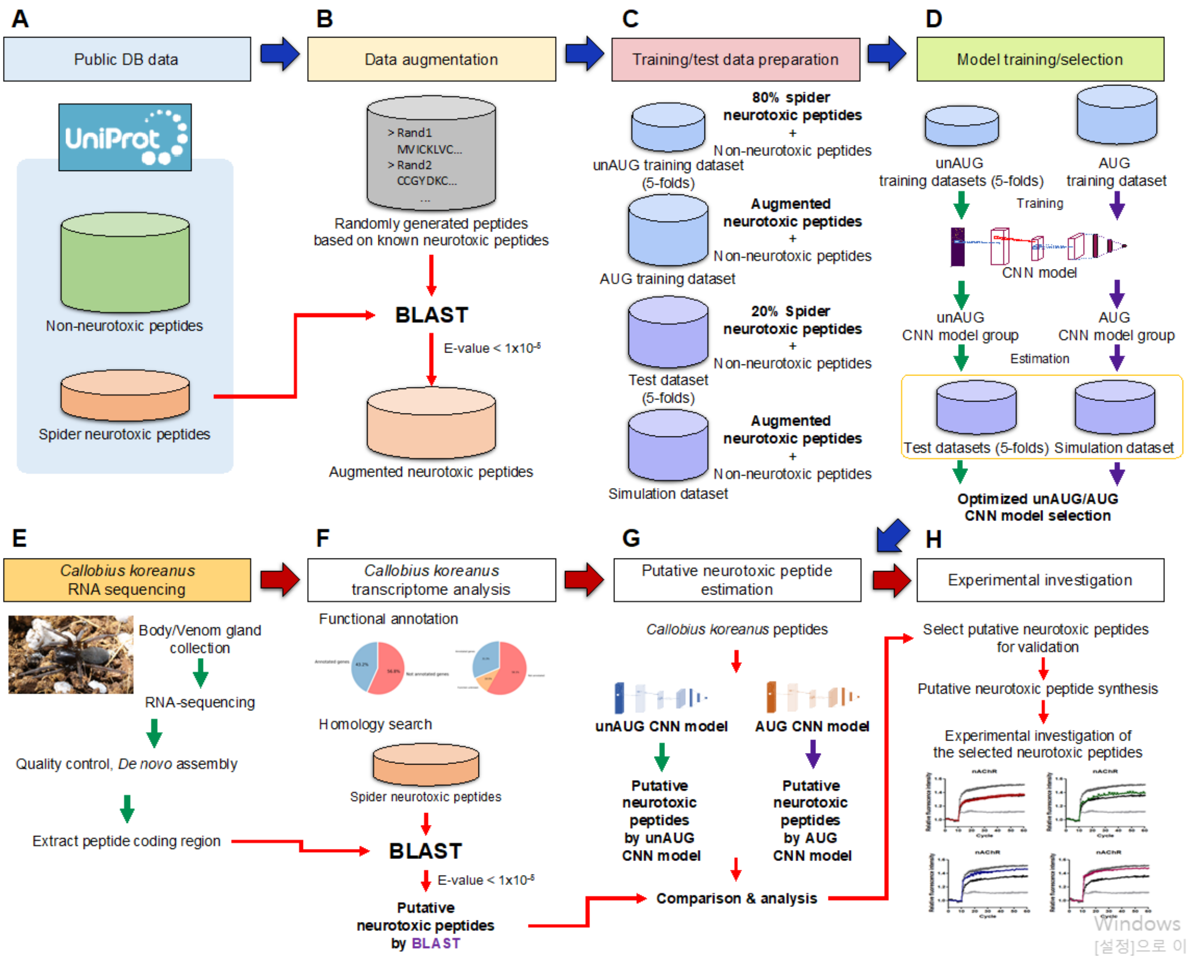

Figure S1. Research workflow. (A) Data for model training and test were obtained from UniProt. (B) Neurotoxic peptide data were augmented with the sequences that were randomly generated and filtered by BLAST search. (C) Two types for each training and test datasets were organized. Each AUG and unAUG training dataset only included known neurotoxic peptides or augmented neurotoxic peptides. (D) CNN models were trained by two types of training datasets, AUG and unAUG. Performance evaluation was conducted with test and simulation datasets. The optimized CNN models from each model group were selected by F1 score of the simulation dataset. (E) The venom glands of *C. koreanus* were separated from the body, and transcriptome data were generated from these samples. Peptide coding regions were found from the transcriptome. (F) Analyses in search of differentially expressed genes (DEG), functional annotation, and homology were performed using the transcriptome data. BLAST was used for screening of putative neurotoxic peptides from *C. koreanus*. (G) We estimated neurotoxic peptides using two of the selected AUG and unAUG CNN models. These peptides were compared with the BLAST result. (H) Four putative peptides were selected and investigated of the modulatory effect on ion channels.

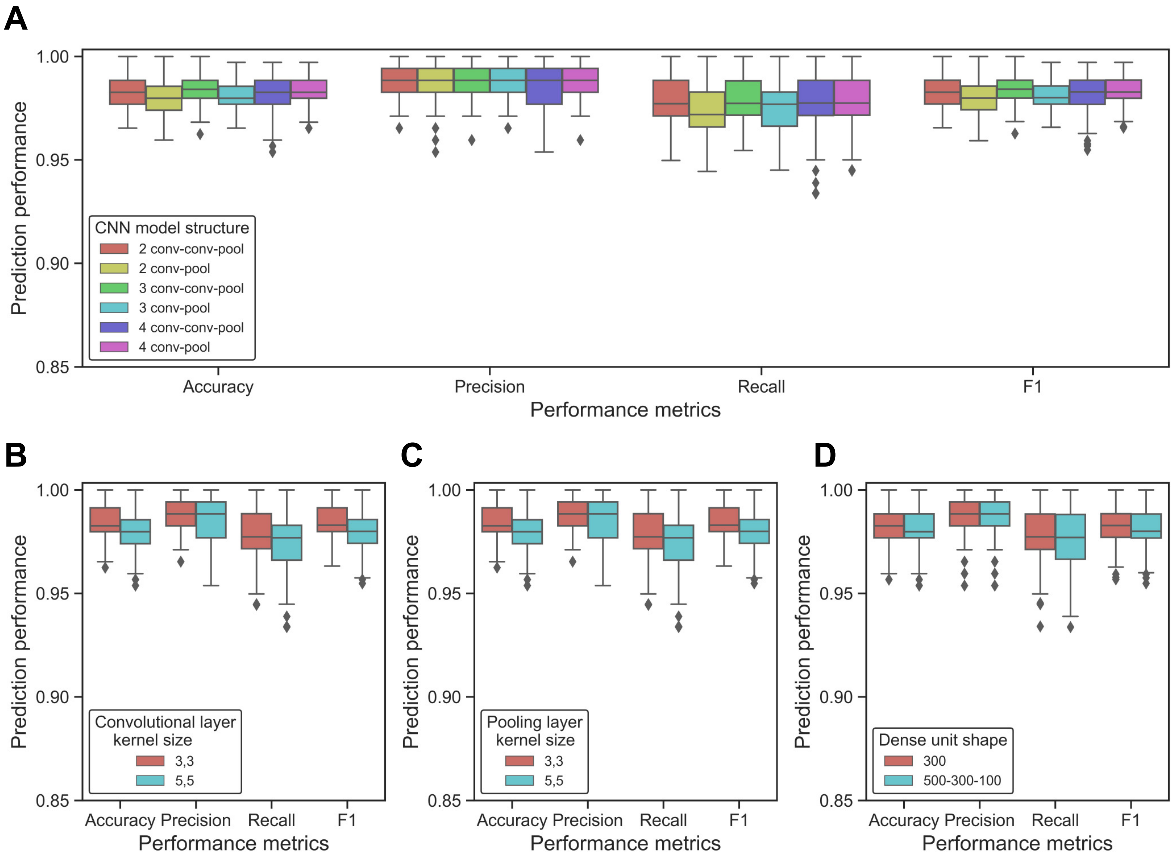

Figure S2. Prediction results from the unAUG CNN model with the test data. (A) Boxplots showing prediction performance comparison according to model structure, (B) convolutional layer kernel size, (C) pooling layer kernel size, and (D) dense layer unit shape.

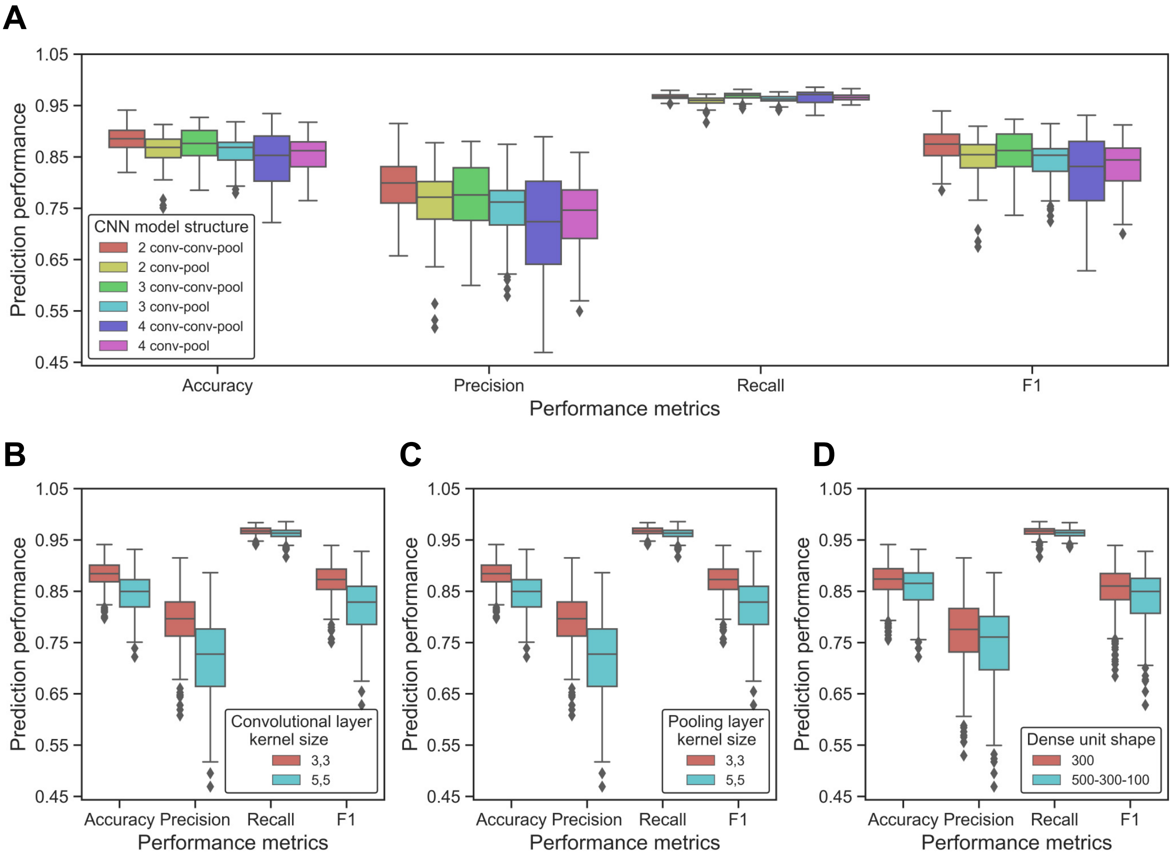

Figure S3. Prediction results from the unAUG CNN model with the simulation data. (A) Boxplots showing prediction performance comparison according to model structure, (B) convolutional layer kernel size, (C) pooling layer kernel size, and (D) dense layer unit shape.

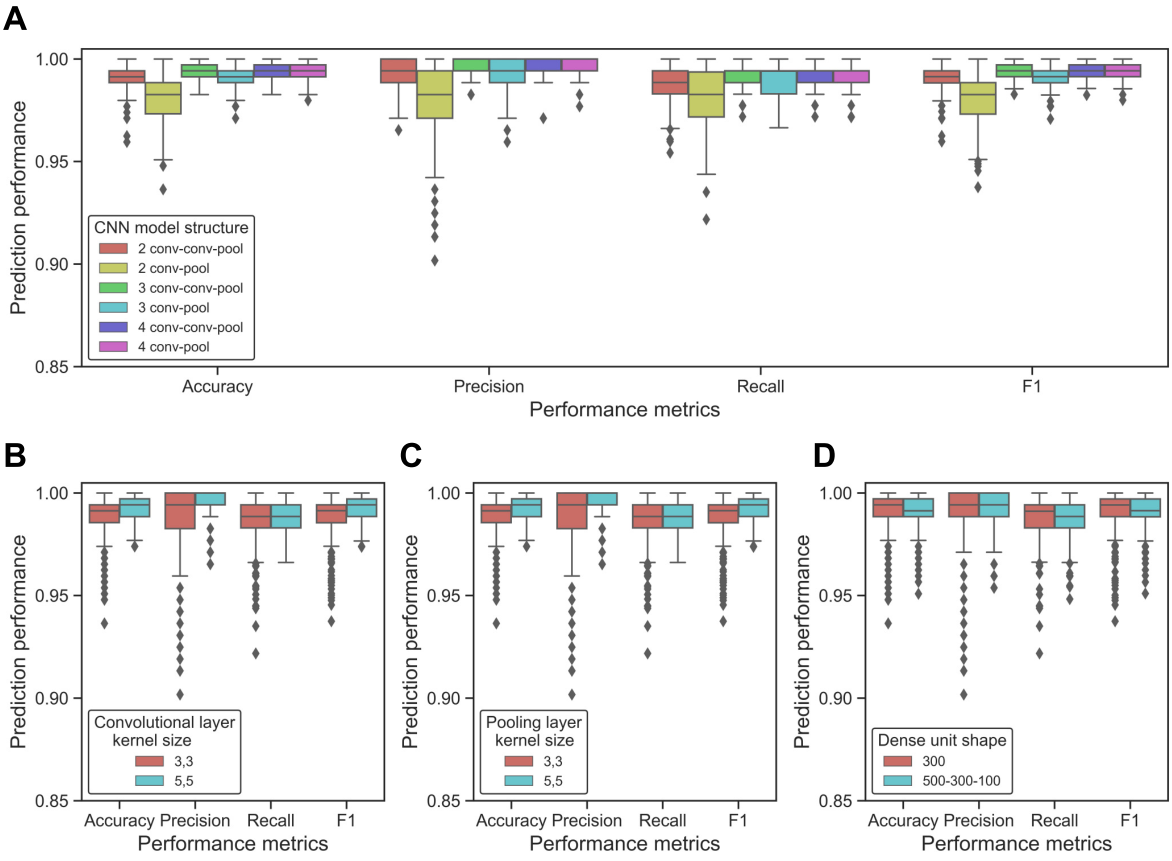

Figure S4. Prediction results from the AUG CNN model group with the test data. (A) Boxplots showing prediction performance comparison according to model structure, (B) convolutional layer kernel size, (C) pooling layer kernel size, and (D) dense layer unit shape.

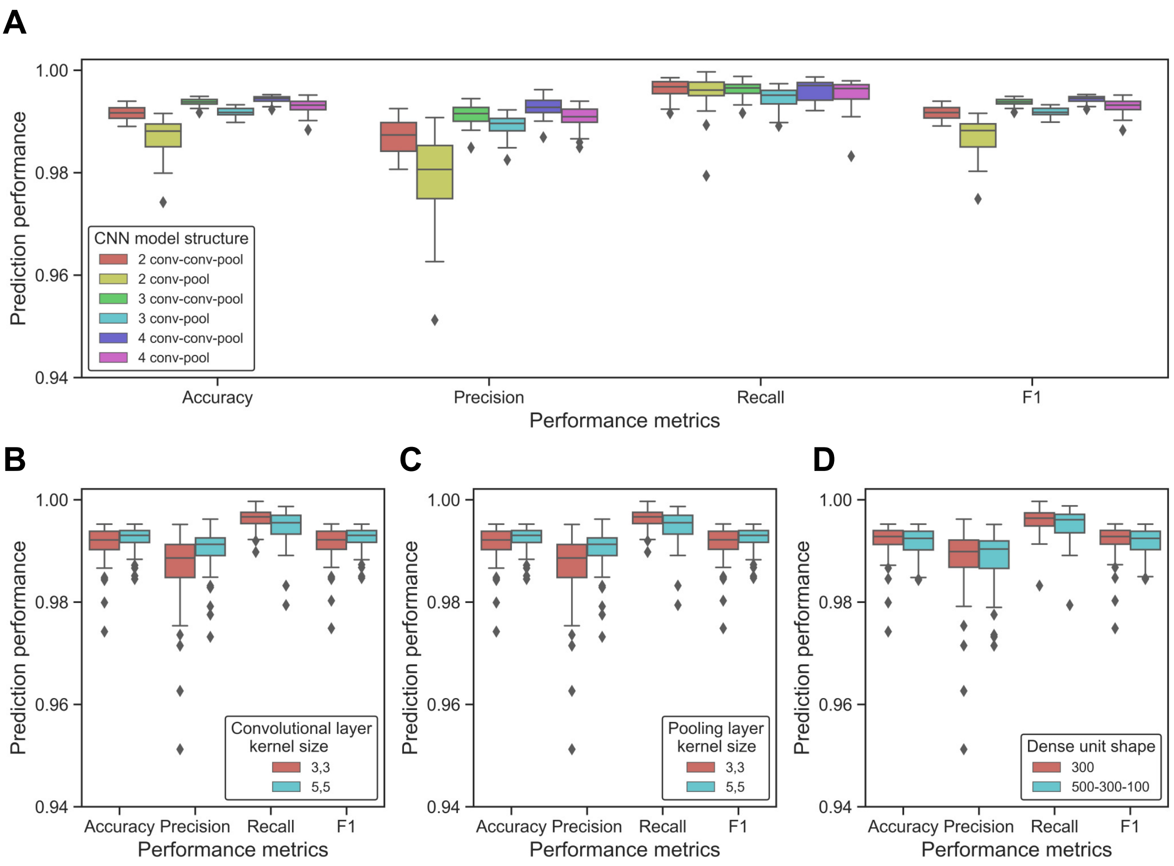

Figure S5. Prediction results from the AUG CNN model with the simulation data. (A) Boxplots showing prediction performance comparison according to model structure, (B) convolutional layer kernel size, (C) pooling layer kernel size, and (D) dense layer unit shape.

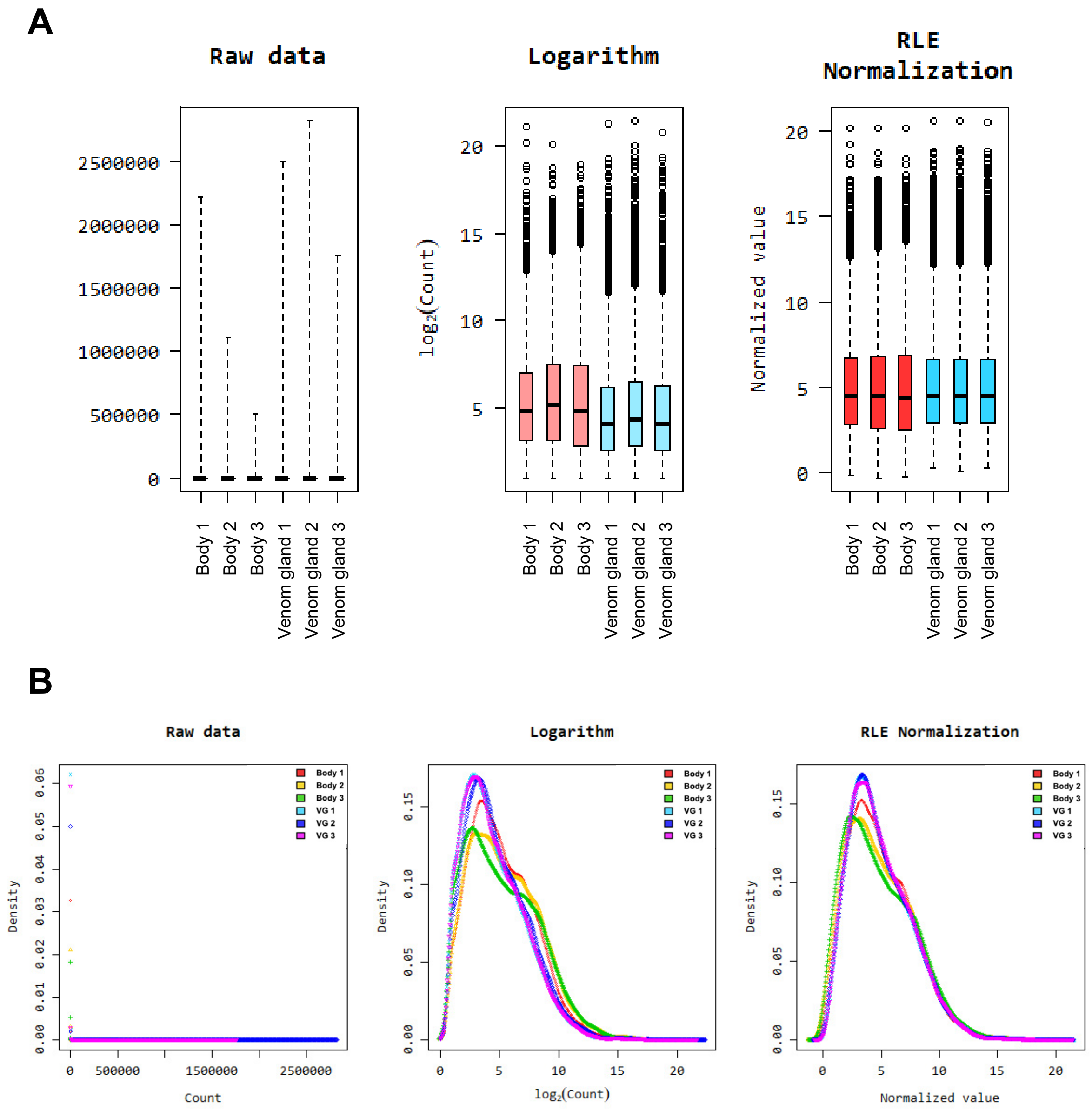

Figure S6. Preprocessing of *C. koreanus* transcriptome data. (A) The distribution of the transcriptome data before and after adjusting for batch effects. *C. koreanus* transcriptome read counts of raw data, log scaled, and relative log expression (RLE) normalization were shown in boxplots. (B) Density plots showing *C. koreanus* transcriptome read counts of raw data, log scaled, and RLE normalization.

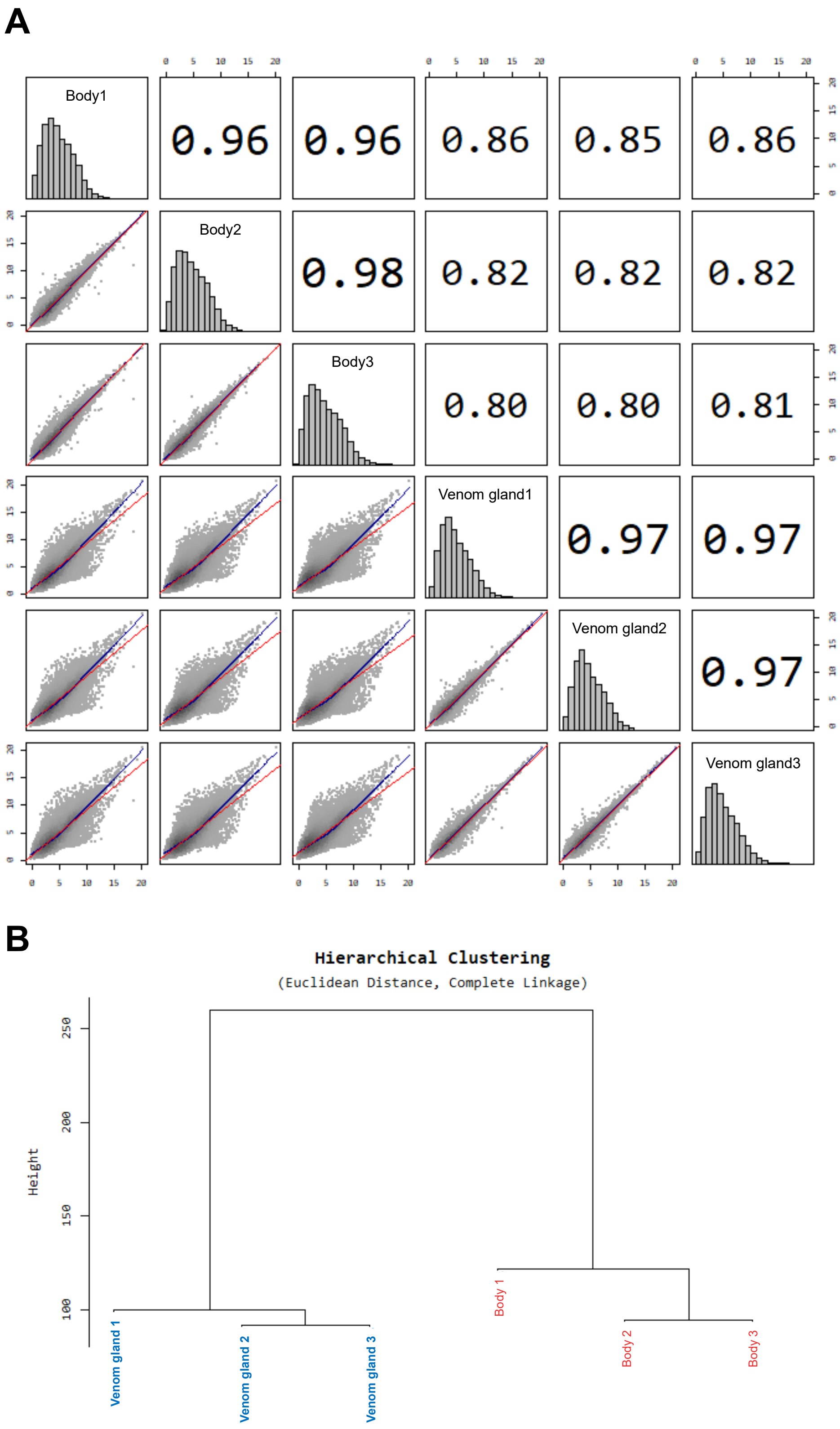

Figure S7. Reproducibility among *C. koreanus* samples. (A) Sample reproducibility was checked by scatterplots, Pearson correlation, and density of each pair of samples. Each information was shown on lower triangular, upper triangular, and diagonal, respectively. (B) A sample dendrogram showing hierarchical clustering of each sample.

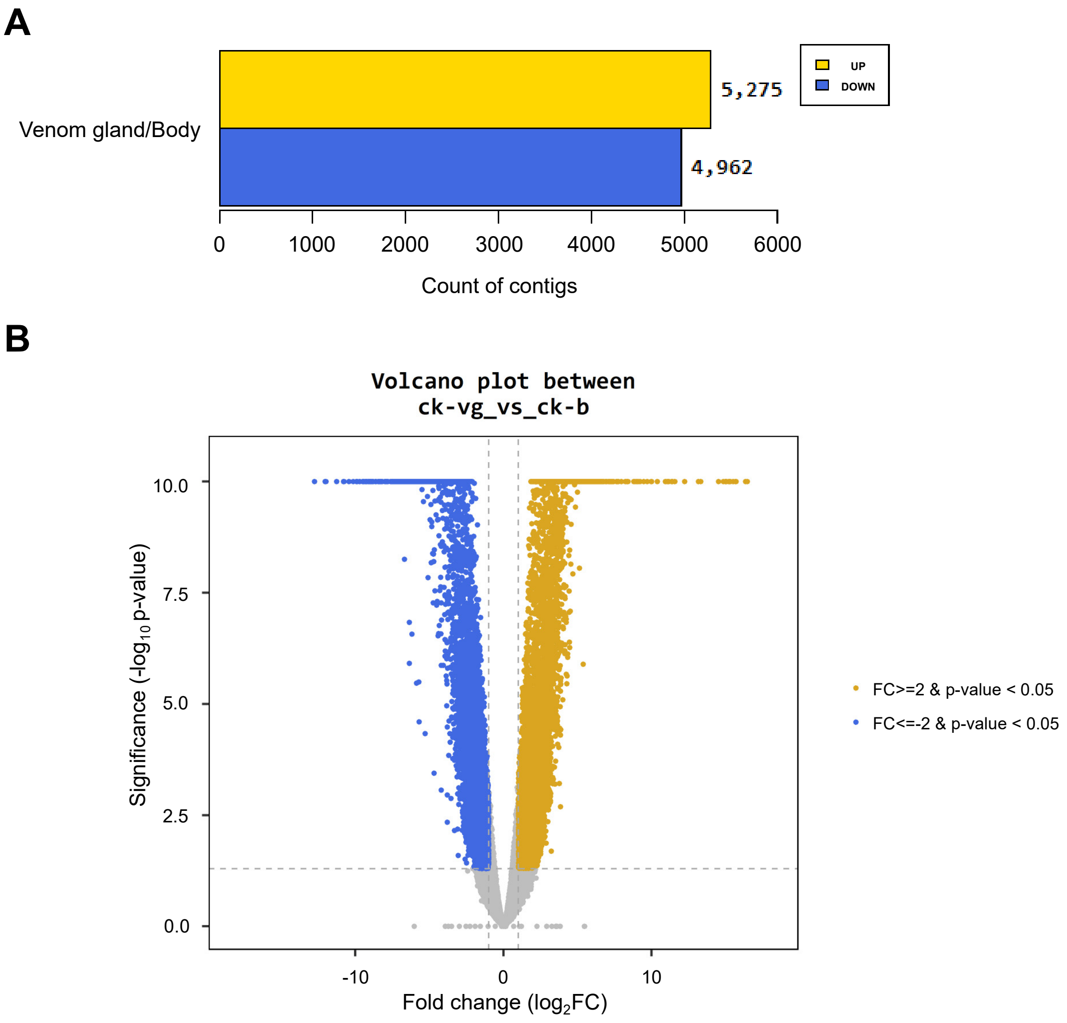

Figure S8. DEG analysis of *C. koreanus* transcriptome data. (A) Significant up and down contigs with criteria ∣fold change∣>=2 and P-value<0.05 were shown. (B) A volcano plot highlighting significant contigs.

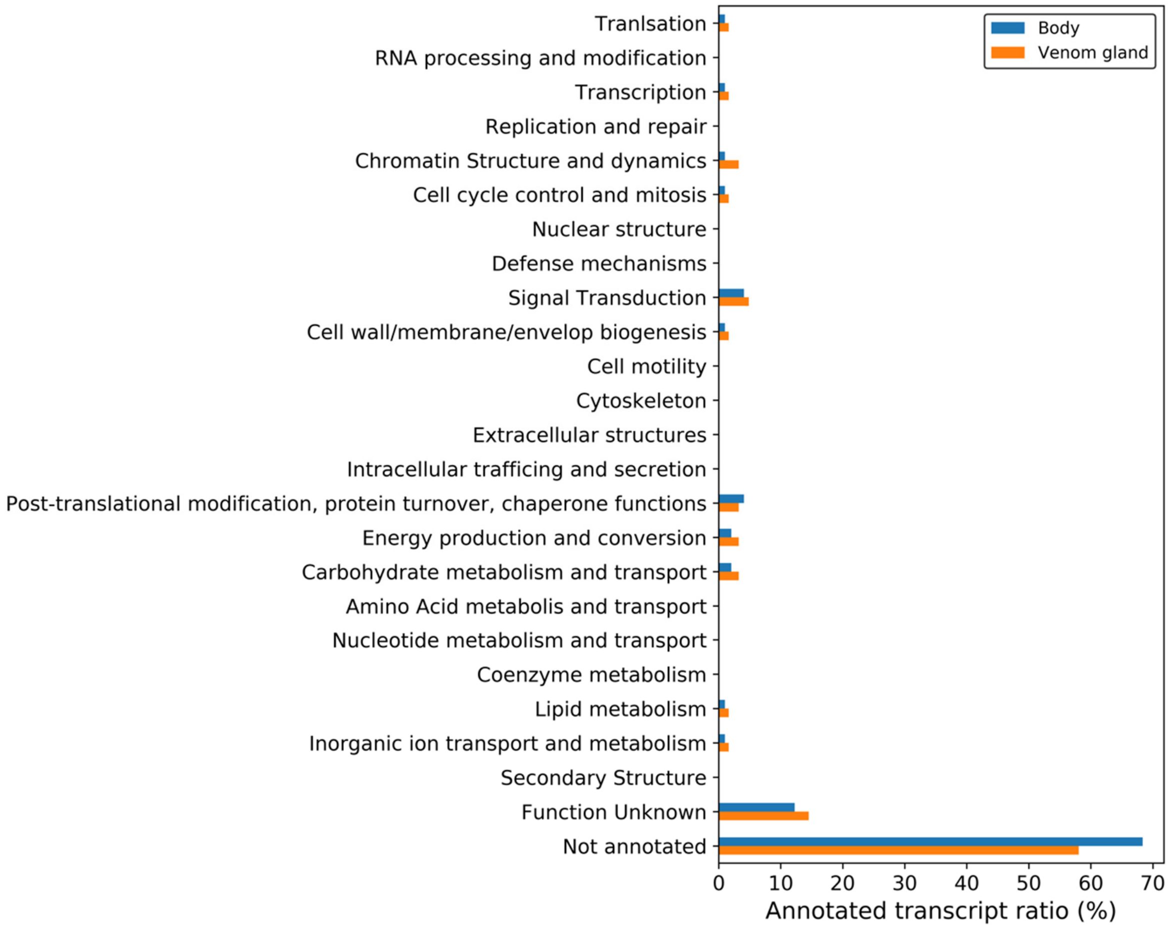

Figure S9. Functional annotation of the clusters of orthologous groups (COGs) from *C. koreanus* peptide-coding genes.

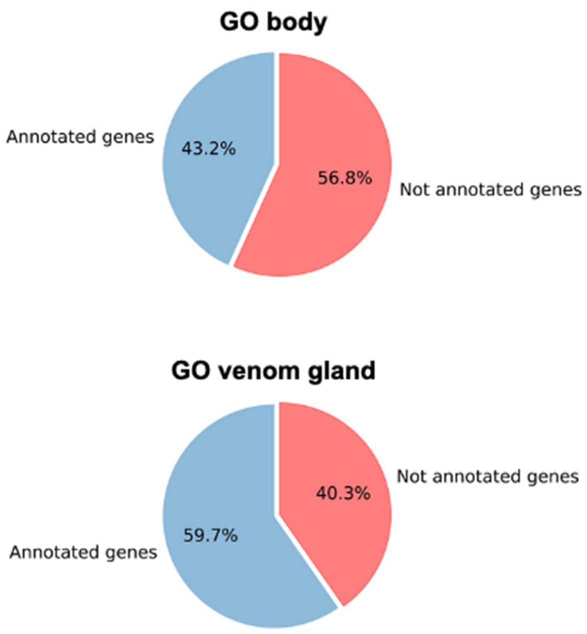

Figure S10. Functional annotation of gene ontology (GO) from *C. koreanus* peptide-coding genes. The annotation results of GO from the body (top) and the venom gland (bottom) were shown.

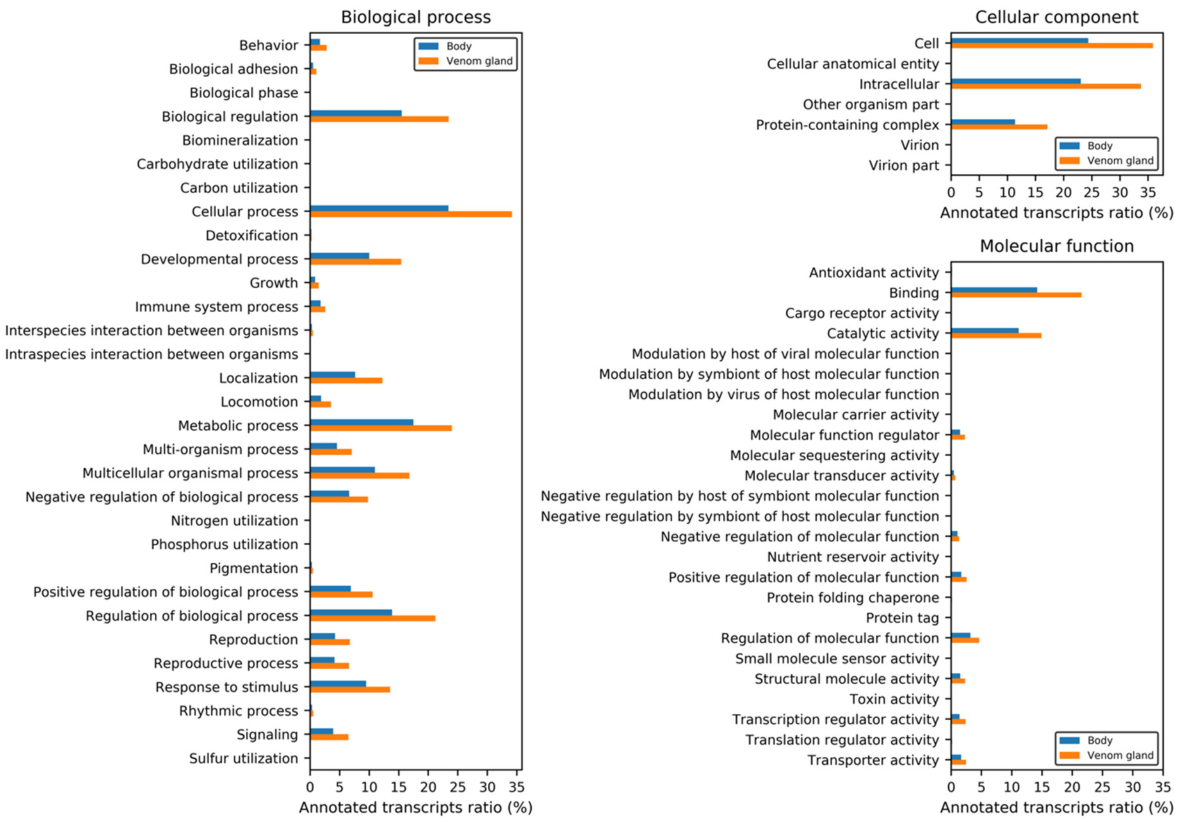

Figure S11. Distribution of the level-2 GO terms of *C. koreanus* peptide-coding genes. Annotation results of GO terms (Level 2) from the body and venom gland were shown with an annotated transcripts ratio.

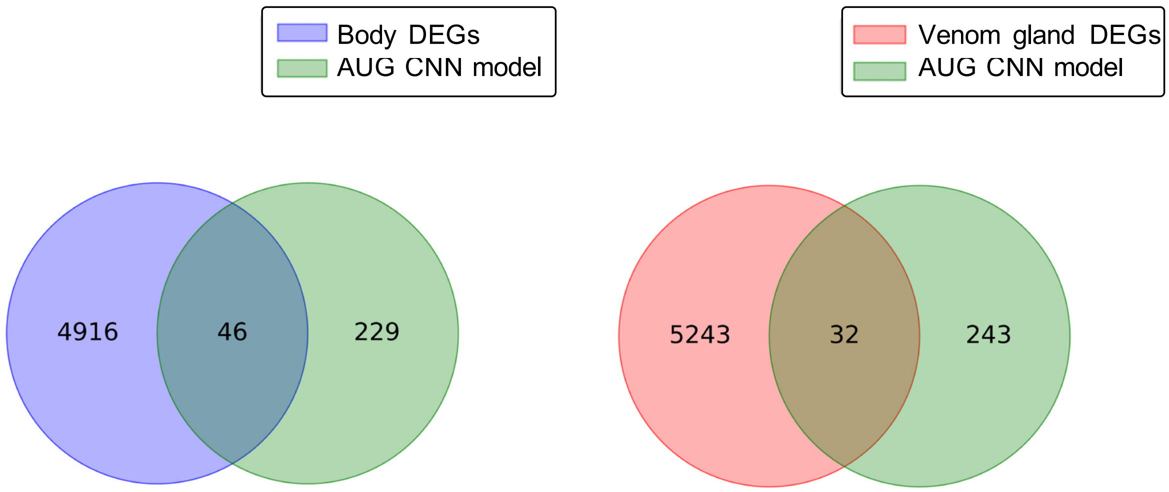

Figure S12. Comparison of *C. koreanus* transcriptome between the DEGs and prediction results from the AUG CNN model. Estimated neurotoxic peptides by the AUG model were presented along with the DEG results of body (left) and venom gland (right).

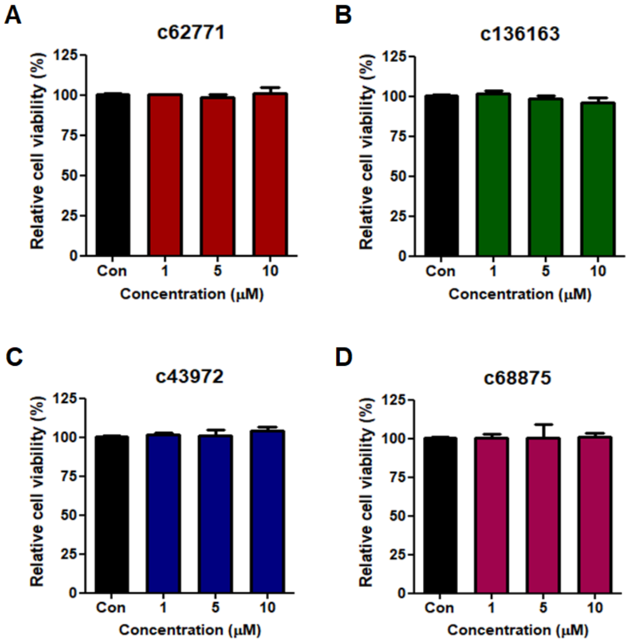

Figure S13. Evaluation of cytotoxicity of the peptides against human neuroblastoma cell line. Treatment of peptides (A) c62771, (B) c136163, (C) C43972, and (D) c68875 had no significant effect on SH-SY5Y cell viability at the concentration of 1, 5, 10 μM.
